# Are lipid droplets the picnic basket of brain tumours?

**DOI:** 10.1038/s41420-024-01797-8

**Published:** 2024-01-16

**Authors:** Tanmayi Bingi, Kian Cotton, Charley Comer, Maria Victoria Niklison-Chirou

**Affiliations:** 1 Life Sciences Department, University of Bath, Claverton Down, Bath, BA2 7AY UK; 2 Centre for Therapeutic Innovation, Life Sciences Department, University of Bath, Claverton Down, Bath, BA2 7AY UK

**Keywords:** Cancer metabolism, Cancer metabolism

## Abstract

Are lipid droplets (LDs) necessary to maintain the viability of brain tumour cells as they move to new nutrient-poor environments? In turn, could cancers be targeted by attacking what you might think of as the cancer cells’ picnic basket? Lipid metabolism reprogramming, represented by increased lipid uptake, activation of de novo lipogenesis and increased lipid storage, is a newly identified hallmark of cancers. Recently, the presence of lipid droplets has been detected in several types of cancers, such as metastatic hepatocellular carcinoma, pancreatic and breast. LDs are storage organelles that provide a source of nutrients which may drive metastasis in different tumours. Currently, several roles of LDs have been posited in various tumours. This perspective aims to review and discuss the currently understood role of LDs in brain tumours.

## Introduction

**Fig. 1:**
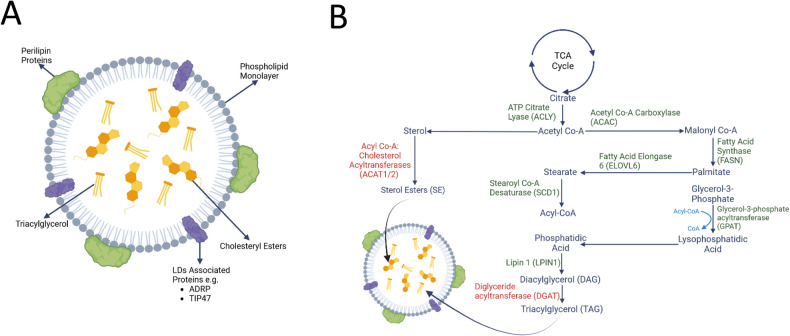
Structure and formation mechanisms of LDs. **A** Illustration depicting the structural composition of LDs. These droplets consist of a central hydrophobic core containing neutral lipids, primarily triacylglycerols (TAGs) and sterol esters. Surrounding this core is a monolayer of phospholipids, incorporating various proteins such as perilipin and lipid droplet-associated proteins (ADRP, TIP47). **B** Diagram outlining the fatty acid synthesis pathway responsible for generating triacylglycerols (TAG) and sterol esters stored within lipid droplets. TAG synthesis occurs on the endoplasmic reticulum membrane, involving sequential addition of fatty acids (FAs) in their activated acyl-CoA form to a glycerol-3-phosphate (G3P) backbone. This process yields lysophosphatidic acid (LPA), phosphatidic acid (PA), and diacylglycerol (DAG). Catalyzed by acyltransferase enzymes, including glycerol-3-phosphate acyltransferases (GPATs), acylglycerol-3-phosphate acyltransferases (AGPATs), and phosphatidic acid phosphatases (lipins), the final and pivotal step in the TAG synthesis cascade is carried out by diacylglycerol acyltransferase enzymes (DGATs). Figure created using BioRender.com.

Lipid droplets (LDs) are dynamic organelles within the cytoplasm, rich in lipids, that play a role in storage and break down of neutral lipids. They are predominantly localised in adipose tissue [1]. LDs are spherical with a core composed of triacylglycerols (TAG) and cholesteryl esters serving as a reservoir for membrane formation and energy homeostasis (Fig. 1A) [2]. Interestingly, LDs can be identified in non-adipose tissues, however, their role and significance, both at homeostasis and in the pathology of various diseases, is still a matter of debate. In such non-adipose tissues, LDs protect against lipotoxicity, a type of cell death induced by the accumulation of excess lipids [2]. Furthermore, it has been reported that LDs in non-adipose tissues are a deposit of signalling precursors and vitamins, aiding protein turnover whilst protecting from oxidative stress [3]. Of interest, literature suggests that LDs tend to increase during cellular differentiation [4–6] and have been detected in various brain conditions such as Alzheimer’s and Parkinson’s disease [7]. Thus, highlighting the diverse potential functions of these dynamic organelles.

As the most lipid-rich organ, the brain utilises lipid metabolism extensively for energy homeostasis, but it also plays a role in neuroinflammation and oxidative stress [8]. Therefore, we ask how much of what we know currently about the role of LDs in healthy adipose and non-adipose, such as stem cells, could be translated into a brain tumour.

Reprogramming of lipid metabolism is a recently recognized characteristic of cancer, where tumour cells increase both their production of new lipids and uptake of fatty acids [9]. This, therefore, increases intracellular lipid storage, reflected as an accumulation of LDs which can be catabolised to generate energy, thus, supporting growth, survival, and migration [10–14]. Recent publications have identified that glioblastoma tumours use LDs to grow under glucose-restricted conditions [1, 15]. Numerous potential roles of LDs have been identified in different cancers, such as enhancing cell proliferation, metastasis, chemotherapy resistance and crosstalk with the immune system [11–14]. Within the central nervous system (CNS), lipids utilised in the formation of LDs can be derived from de novo lipogenesis, lipoprotein particles, apoptotic cells, debris, myelin, neuron-released lipids and synaptic pruning, among other sources [16].

LDs formation occurs at the endoplasmic reticulum (ER) and involves a complex series of steps (Fig. 1B) [2]. Firstly, activated fatty acids (FAs) are esterified to diacylglycerol or sterol to form neutral lipids [2]. The formation of both compounds involves several enzyme-regulated steps (Fig. 1B). Most pertinent of these are, Acyl-CoA: cholesterol O-transferases (ACAT1/2), which catalyse the formation of sterol esters (SEs) and diacylglycerol acyltransferases (DGAT1/2), which catalyse the formation of triacylglycerols (TAGs) [2]. As the concentration of the neutral lipids increases, they form an oil lens by depositing between the inner and outer leaflets of the ER [2]. The oil lens expands, leading to LDs budding from the ER membrane [2]. This is facilitated by serpin, a class I protein which allows lipids to move into the LDs during budding [1, 2]. Class II binding proteins such as the perilipins-1-5 (PLIN1-5) protect LDs from hydrolysis by lipases such as adipose triglyceride lipase (ATGL) [1, 2].

**Fig. 2:**
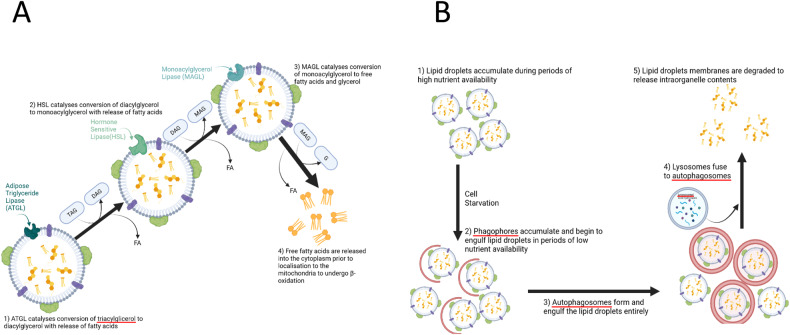
Dynamic processes in LDs breakdown. LDs breakdown is orchestrated through two primary mechanisms: lipolysis and lipophagy. **A** Schematic illustration delineating TAG intracellular lipolysis. This process unfolds in a sequential cascade involving adipose triglyceride lipase (ATGL), hormone-sensitive lipase (HSL), and monoacylglycerol lipase (MAGL), all situated on the LDs surface. ATGL initiates the process, catalyzing the conversion of triacylglycerols (TAGs) into diacylglycerol (DAG) and fatty acids (FAs). Subsequently, HSL further breaks down DAG into monoacylglycerol (MAG) and FAs. Finally, MAGL liberates FAs and glycerol. The lipolytic products, including DAG, MAG, FAs, and glycerol, serve as energy resources, membrane biosynthesis and others. **B** Lipophagy, an intricately regulated form of autophagy, engages the encapsulation of lipid droplets within autophagosomal membranes. These engulfed droplets are subsequently transported to lysosomes, where degradation occurs through acid lipolysis. Lysosomal TAG lipolysis is orchestrated by lysosomal acid lipase (LAL). Figure crafted using BioRender.com.

LDs catabolism is the process by which LDs are broken down to mobilize neutral lipid stores during periods of nutrient starvation or intense energy demand such as high rates of proliferation [2]. Two main breakdown pathways exist: lipophagy (Fig. 2A) and lipolysis (Fig. 2B) [2]. During lipolysis, lipases attach to the surface of lipid droplets and break down TAGs into FAs and glycerol (Fig. 2A). The breakdown of TAGs in lipolysis occurs through the step-by-step process involving adipose triglyceride lipase (ATGL), hormone-sensitive lipase (HSL), and monoacylglycerol lipase (MAGL) (Fig. 2A) [14]. In the process of lipophagy, a type of autophagy, LDs are engulfed and merge with lysosomes, where they are broken down by lysosomal acid lipase (LAL) (Fig. 2B) [17].

It has been recently documented that LDs play an important role in cancer [9–15]. Tumour cells are exposed to a dynamic environment where nutrient and oxygen levels fluctuate between the core and periphery of the tumour mass [9, 10]. As a direct result, cancer cells utilise multiple metabolic pathways to obtain energy and survive and proliferate [9, 10]. They maintain their lipid supply by increasing de novo lipogenesis and recycling structural lipids by autophagy or enzymatic remodelling [18]. This lipid supply is incorporated in LDs that, when required, are used to generate ATP, regulate autophagy, maintain redox balance and synthesise cell membranes [18, 19]. During tumour progression, this reduces the cellular stress of energy demand, allowing further development of the tumour [19].

We suggest that brain tumour cells accumulate LDs, which essentially act as a ‘picnic basket’, supplying an energy source for tumour progression during periods of environmental and cellular stress. Glioblastoma multiforme (GBM) is a prevalent and highly invasive brain tumour found in adults [15, 19]. In a study comparing fast-cycling (FC) and slow-cycling (SC) cells present in GBM tumours, it was found that SC cells displayed greater levels of lipid metabolites derived from LDs when exposed to low glucose conditions [15]. Thus, implicating a role for LDs as an energy reserve in SC cells [15]. SC cells in GBM also demonstrated a more invasive and migratory phenotype than FC cells [15]. Another study investigated the link between hyperactivation of mammalian target of rapamycin complex (mTORC)-1 and lipophagy in GBM cells [19]. mTORC1 integrates growth factor signals, nutrients and available energy to drive cell growth and catabolism [20]. It was shown that GBM cells sustain their energy production through lipophagy of LDs. Furthermore, use of the glycolysis inhibitor, 2-Deoxy-D-Glucose, correlated with hyperactivation of mTORC, linked with poor prognoses in GBM [19]. Yang K et al. (2021) discovered a physical interaction between choline kinase α2 (CKα2) and LDs [21]. AMP-activated protein kinase (AMPK) induces post-translational modifications at lysine247 through phosphorylation of CKα2 when GBM cells become starved of glucose, an action mediated by lysine acetyltransferase 5 [21]. This enables CKα2 to function as a protein kinase, phosphorylating PLIN2/3 on the surface of LDs, resulting in lipolysis [21]. Therefore, it appears that oxidation of FAs released from LDs fuels GBM, highlighting its role as a ‘picnic basket’ in brain tumour cells. Interestingly, there may also be a central role for LDs in stem cell fate determination [4]. Feng Yue et al., found that LDs accumulated in stem cells that are committed to differentiation rather than self-renewal. This suggests that LDs are linked with the differentiation mechanism of stem cells, although their role in stem cells fate remains ambiguous.

Based on the evidence discussed here and in published literature on different cancers, it can be concluded that one of the primary functions of LDs in brain tumours is to be the ‘picnic basket’. As a fully modulatory energy source, they are able to support energy-intense processes such as cell proliferation and in turn, drive aggressive phenotypes.

Moreover, it is not without reason to posit that these organelles may play a key role in supporting or facilitating metastatic phenotypes within tumours. The complex role of LDs in cancer and other non-adipocyte cells remains largely uncharacterised. We believe that the different processes involving lipid mobilization from these dynamic organelles can be developed as a new strategy for the treatment of brain tumours.
